# Secretory Proteome of Brown Adipocytes in Response to cAMP-Mediated Thermogenic Activation

**DOI:** 10.3389/fphys.2019.00067

**Published:** 2019-02-07

**Authors:** Joan Villarroya, Rubén Cereijo, Marta Giralt, Francesc Villarroya

**Affiliations:** ^1^Departament de Bioquímica i Biomedicina Molecular and Institut de Biomedicina (IBUB), Universitat de Barcelona, Barcelona, Spain; ^2^Infectious Diseases Unit, Hospital de la Santa Creu i Sant Pau, Barcelona, Spain; ^3^CIBER Fisiopatología de la Obesidad y Nutrición, Madrid, Spain

**Keywords:** brown adipose tissue, thermogenesis, secretome, adipokine, extracellular matrix

## Abstract

**Background:** The secretory properties of brown adipose tissue are thought to contribute to the association between active brown fat and a healthy metabolic status. Although a few brown adipokines have been identified, a comprehensive knowledge of the brown adipose tissue secretome is lacking.

**Methods:** Here, to examine the effects of thermogenic activation of brown adipocytes on protein secretion, we used isobaric tags for relative and absolute quantification (iTRAQ) analysis to determine how the secreted proteome of brown adipocytes (that detected in cell culture medium) differed in response to cAMP.

**Results:** Our results indicated that 56 secreted proteins were up-regulated in response to cAMP. Of them, nearly half (29) corresponded to extracellular matrix components and regulators. Several previously known adipokines, were also detected. Unexpectedly, we also found five components of the complement system. Only 15 secreted proteins were down-regulated by cAMP; of them three were ECM-related and none was related to the complement system. We observed a partial concordance between the cAMP-regulated release of proteins (both from proteomics and from antibody-based quantification of specific proteins) and the cAMP-mediated regulation of their encoding transcript for the up-regulated secreted proteins. However, a stronger concordance was seen for the down-regulated secreted proteins.

**Conclusions:** The present results highlight the need to investigate previously unrecognized processes such as the role of extracellular matrix in thermogenic activation-triggered brown fat remodeling, as well as the intriguing question of how brown adipocyte-secreted complement factors contribute to the signaling properties of active brown adipose tissue.

## Introduction

The high capacity of brown adipose tissue (BAT) for energy expenditure and oxidation of glucose and lipids is associated with protection against obesity, hyperglycemia and hyperlipidemia in rodents and possibly in humans (Bartelt et al., 2011; Giralt and Villarroya, 2017; Carobbio et al., 2018). However, the effects of BAT on metabolism may also reflect the release of bioactive factors that act in autocrine, paracrine, and even endocrine manners (Villarroya et al., 2017). The secretory activity of white fat has long been recognized and a large number of “white” adipokines have been identified. In contrast, the secretory properties of BAT have received much less research attention. Most brown adipokines were identified from the high-level expression of genes encoding putative secreted proteins, such as fibroblast growth factor-21 (FGF21) or interleukin-6 (IL-6) in active BAT (Burýsek and Houstek, 1997; Hondares et al., 2011). In some cases, brown adipokine candidates were identified by screening transcriptomic data from BAT using bioinformatic tools that can predict the “secretability” of encoded factors (Wang et al., 2014; Verdeguer et al., 2015; Cereijo et al., 2018). Other approaches have used indirect methodologies, such as signal sequence trap (Hansen et al., 2014). However, we still lack a comprehensive knowledge of the BAT secretome. Here, we use an updated iTRAQ-based proteomic analysis to provide the first report of a direct assessment of proteins secreted by brown adipocytes in response to cAMP, which is the main intracellular mediator of BAT thermogenic activity.

## Materials and Methods

### Cell Culture

Murine C57BAT brown pre-adipocytes were supplied by Klein et al. (1999) and were obtained using protocols approved by the Institutional Animal Care and Use Committee, Joslin Diabetes Center (Boston, USA). Pre-adipocytes were cultured in DMEM (Life Technologies, Grand Island, NY, USA), 10% FBS, 1% penicillin-streptomycin, for 2–3 days. Before differentiation was induced, the plates were rinsed with PBS to remove the FBS; further culture was performed in FBS-free medium. Differentiation was induced by culturing cells overnight in DMEM plus T3 (1 nM), insulin (20 nM), IBMX (500 μM), dexamethasone (2 mM), and indomethacin (125 μM), and then replacing this medium with DMEM plus T3 and insulin until brown adipocytes had fully differentiated (5–6 days). Differentiated brown adipocytes were left untreated or treated with 1 mM dibutyryl-cAMP for 24 h. Three independent samples were used for each group. Medium was collected and cells were scrapped and collected for RNA analysis. Unless otherwise noted, reagents were from Merck Sigma-Aldrich, S. Louis, MO, USA.

### Comparative Proteomic Analysis iTRAQ

Identification of proteins with differences in abundance between cAMP-treated and non-cAMP-treated brown adipocytes was performed using the isobaric tag for relative and absolute quantitation (iTRAQ) technology (see Supplementary Material for detailed methodology). Three independent replicates were prepared for each sample. Samples were resuspended in 500 mM triethyl ammonium bicarbonate/8M urea solution. Proteins were reduced with tris (2-carboxyethyl) phosphine, alkylated with iodoacetamide and digested with porcine trypsin (Promega, Madison, WI, USA). Digested peptides were labeled with 8-plex iTRAQ reagent (AB Sciex, Wien, Austria). Pooled peptides were cleaned up in two steps: C18 clean-up (reverse phase, toptip C18) and then SCX cleanup (strong cationic exchange, P200 toptip, PolySULFOETHYL A) (PolyLC, Columbia, MD, USA). Peptides were analyzed by liquid chromatography coupled to mass spectrometry using nanoAcquity (Waters, Milford, MA, USA) coupled to LTQ-Orbitrap Velos mass spectrometer (ThermoScientific, Foster City, CA, USA). Up to the 10 most abundant peptides were selected from each MS scan and then fragmented using Higher Energy Collision Dissociation. Database search was performed with Mascot search engine against SwissProt/Uniprot database. IsobariQ software (Arntzen et al., 2011) was used to perform the relative quantitation of proteins by iTRAQ. We used the annotation “extracellular” in the Gene Onthology database, TargetP 1.1 (Emanuelsson et al., 2007), and Secretome P 2.0 (Bendtsen et al., 2004) software to identify secreted proteins. Crude data from proteomics analysis are available at www.adipoplast.org/datebase.

### Quantitative Real Time PCR

RNA was extracted (Nucleospin, Macherey-Nagel, Düren, Germany) and retrotranscribed using TaqMan Reverse Transcription Reagents (Applied Biosystems, Foster City, CA, USA). Transcript levels were determined by quantitative real-time PCR on a 7500 Real-Time PCR System (Applied Biosystems), using TaqMan assays or SyberGreen assays with specifically designed primers (PerlPrimer software) (see Supplementary Material). The mRNA levels of each gene of interest was normalized to 18S rRNA levels using the 2^−Δ*Ct*^ method.

### Specific Quantification of Secreted Proteins

Individual proteins were quantified in brown adipocyte culture medium using specific ELISA kits (see Supplemental Table 2 for suppliers), except retinol-binding protein-4 (RBP4), which was quantified by immunoblotting, as previously described (Rosell et al., 2012).

## Results

### Differentiation and Effects of cAMP in Brown Adipocytes Cultured in Serum-Free Medium

**Figure 1:**
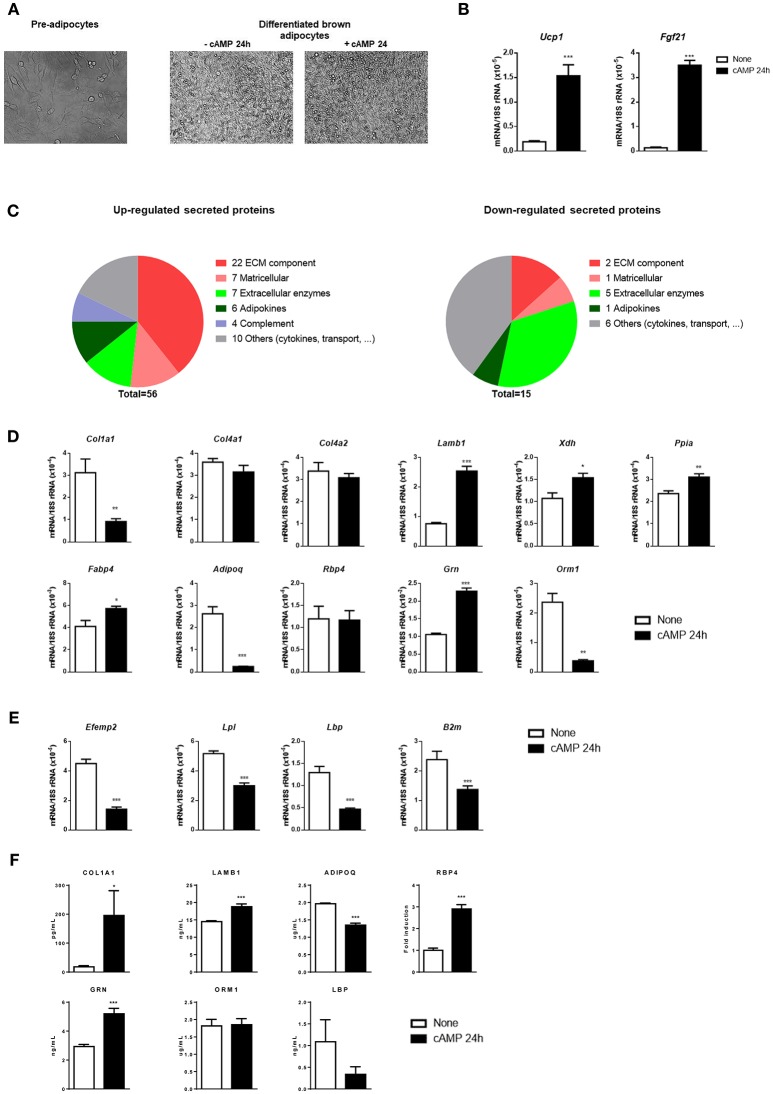
Differentiation, transcript levels, and secreted proteins in cultured brown adipocytes in response to cAMP. **(A)** Representative optical microscopic images of pre-adipocytes (left), and brown adipocytes left untreated or treated with cAMP for 24 h (right). **(B)** Relative transcript levels of *Ucp1* and *Fgf21* in untreated (none) and cAMP-treated (cAMP 24 h) differentiated brown adipocytes (*n* = 6). **(C)** Secreted proteins found to be up-regulated (left) and down-regulated (right) in response to cAMP were classified into six groups according to their function: ECM component, matricellular, extracellular enzymes, adipokines, complement, and others (cytokines, transport, etc). **(D)** Transcript levels corresponding to selected secreted proteins up-regulated by cAMP treatment of brown adipocytes**. (E)** Transcript levels corresponding to selected secreted proteins down-regulated by cAMP treatment of brown adipocytes. **(F)** Levels of secreted proteins in brown adipocyte culture medium individually quantified using specific antibody-based methods. Bars represent means ± s.e.m of six samples per group. Two-tailed unpaired Student's *t-*test was used to compare means (^*^p < 0.05, ^**^p < 0.01, ^***^p < 0.001, cAMP vs. no treatment).

Our brown adipocyte culture protocol, adapted to allow a proteomics-based analysis of the culture medium (see section Materials and Methods), resulted in more than 90% adipogenic differentiation, as assessed by the percentage of cells exhibiting lipid droplet accumulations (Figure 1A), and expression of the brown adipocyte marker gene *Ucp1*. Cell culture media were collected from cAMP-treated and non-treated controls for proteomic analysis. Transcript levels of *Ucp1* and *Fgf21*, which encodes for a thermogenically-regulated secreted protein (Hondares et al., 2011), were strongly induced in cAMP-treated brown adipocytes (Figure 1B). This validates the experimental model used to perform the proteomic analysis.

### Identification of cAMP-Regulated Secreted Proteins

A total of 71 extracellular proteins were found to differ in their abundance in cAMP-treated vs. non-treated cultures (Table 1). Fifty-six secreted proteins were induced by cAMP (Figure 1C, left). Of them, 40% (22 proteins) were components of the extracellular matrix (ECM) and 13% (7 proteins) were matricellular proteins (non-structural proteins that are present in the ECM and play regulatory roles). Among the remaining up-regulated secreted proteins, seven were extracellular enzymes, six were adipokines, and four corresponded to components of the complement system. Finally, we observed up-regulation of 10 varied proteins, including cytokines, transporters, and proteins of unknown function. Notably, fewer secreted proteins were down-regulated by cAMP (Figure 1C, right). Of these 15 proteins, five were extracellular enzymes, three were ECM-related, one was an adipokine, and six formed a heterogeneous group of proteins with multiple or unknown functions.

**Table 1:** List of secreted proteins found to be up-regulated **(A)** or down-regulated **(B)** upon cAMP treatment of brown adipocytes, categorized by their functions.

**Function**	**UniprotKB**	**Gene symbol**	**Description**	**Length**	**cAMP-induced fold-change**
**(A) UP REGULATED PROTEINS IN RESPONSE To cAMP**					
ECM component	P07356	*Anxa2*	Annexin A2	339	1.12
	Q62059-3	*Vcan*	Versican core protein	2,393	1.56
	P97927	*Lama4*	Laminin subunit alpha-4	1,816	1.36
	P02469	*Lamb1*	Laminin subunit beta-1#	1,786	1.51
	P02468	*Lamc1*	Laminin subunit gamma-1	1,607	1.40
	P08122	*Col4a2*	Collagen alpha-2(IV) chain#	1,707	1.36
	Q02788	*Col6a2*	Collagen alpha-2(VI) chain#	1,034	1.33
	P10493	*Nid1*	Nidogen-1#	1,245	1.35
	P82198	*Tgfbi*	Transforming growth factor-beta-induced protein ig-h3	693	1.29
	Q61554	*Fbn1*	Fibrillin-1#	2,871	1.28
	Q3U962	*Col5a2*	Collagen alpha-2(V) chain#	1,497	1.26
	P02463	*Col4a1*	Collagen alpha-1(IV) chain#	1,669	1.25
	P11276	*Fn1*	Fibronectin	2,477	1.25
	P11087	*Col1a1*	Collagen alpha-1(I) chain#	1,453	1.22
	O35206	*Col15a1*	Collagen alpha-1(XV) chain	1,367	1.19
	Q05793	*Hspg2*	Basement membrane-specific heparan sulfate proteoglycan core protein (perlecan)^*^#	3,707	1.18
	P33434	*Mmp2*	72 kDa type IV collagenase	662	1.18
	Q04857	*Col6a1*	Collagen alpha-1(VI) chain#	1,025	1.17
	P08121	*Col3a1*	Collagen alpha-1(III) chain^*^#	1,464	1.12
	O08746-2	*Matn2*	Matrilin-2	956	1.07
	Q61398	*Pcolce*	Procollagen C-endopeptidase enhancer 1^*^#	468	1.07
	O88207	*Col5a1*	Collagen alpha-1(V) chain#	1,838	1.02
ECM matricellular	Q08879	*Fbln1*	Fibulin-1	705	1.19
	P37889-2	*Fbln2*	Fibulin-2 #	1,221	1.20
	Q62009-3	*Postn*	Periostin	838	1.20
	P70663	*Sparcl1*	SPARC-like protein 1	650	1.14
	P07214	*Sparc*	SPARC^*^	302	1.10
	P16110	*Lgals3*	Galectin-3	264	1.11
	Q07797	*Lgals3bp*	Galectin-3-binding protein	577	1.17
Extracellular enzymes	Q8BND5-2	*Qsox1*	Sulfhydryl oxidase 1	748	1.56
	P10605	*Ctsb*	Cathepsin B#	339	1.37
	P00687	*Amy1*	Alpha-amylase 1	511	1.04
	P00688	*Amy2*	Pancreatic alpha-amylase	508	1.13
	P21460	*Cst3*	Cystatin-C#	140	1.10
	Q00519	*Xdh*	Xanthine dehydrogenase/oxidase	1,335	1.07
	P17742	*Ppia*	Peptidyl-prolyl cis-trans isomerase A#	164	1.04
Adipokines	Q60994	*Adipoq*	Adiponectin#	247	1.31
	Q00724	*Rbp4*	Retinol-binding protein 4	201	1.31
	P11859	*Agt*	Angiotensinogen#	477	1.13
	P04117	*Fabp4*	Fatty acid-binding protein, adipocyte	132	1.02
	P03953-2	*Cfd*	Complement factor D (adipsin)#	259	1.01
	Q9DD06	*Rarres2*	Retinoic acid receptor responder protein 2 (Chemerin)^*^#	162	1.01
Complement	P01029	*C4b*	Complement C4-B	1,738	1.09
	P04186	*Cfb*	Complement factor B	761	1.06
	P48759	*Ptx3*	Pentraxin-related protein PTX3	381	1.06
	P01027	*C3*	Complement C3#	1,663	1.05
Others (cytokines, transport,…)	P21956-2	*Mfge8*	Lactadherin	463	1.60
	Q62356	*Fstl1*	Follistatin-related protein 1^*^#	306	1.34
	P28798	*Grn*	Granulins	589	1.24
	P17515	*Cxcl10*	C-X-C motif chemokine 10	98	1.20
	O88968	*Tcn2*	Transcobalamin-2	430	1.14
	Q9Z0J0	*Npc2*	Epididymal secretory protein E1 (Niemann-Pick)^*^#	149	1.09
	P47879	*Igfbp4*	Insulin-like growth factor-binding protein 4^*^#	254	1.09
	O88393	*Tgfbr3*	Transforming growth factor beta receptor type 3 (beta-glycan)#	850	1.08
	Q60590	*Orm1*	Alpha-1-acid glycoprotein 1 (orosomucoid)#	207	1.06
	P97298	*Serpinf1*	Pigment epithelium-derived factor (Serpin1)#	417	1.05
**(B) DOWN-REGULATED PROTEINS IN RESPONSE TO cAMP**					
ECM component Matricellular Extracellular enzymes	P28653	*Bgn*	Biglycan^*^#	369	1.03
	Q8BPB5	*Efemp1*	EGF-containing fibulin-like extracellular matrix protein 1^*^	493	1.35
	P16045	*Lgals1*	Galectin-1	135	1.13
	Q06890	*Clu*	Clusterin	448	1.01
	P10639	*Txn*	Thioredoxin	105	1.07
	P06745	*Gpi*	Glucose-6-phosphate isomerase (neuroleukin)	558	1.06
	P11152	*Lpl*	Lipoprotein lipase^*^	474	1.06
	P18242	*Ctsd*	Cathepsin D	410	1.04
Adipokines Others (cytokines, transport,…)	Q61805	*Lbp*	Lipopolysaccharide-binding protein	481	1.05
	Q61646	*Hp*	Haptoglobin^*^	347	1.15
	P13020	*Gsn*	Gelsolin#	780	1.07
	Q61207	*Psap*	Prosaposin	557	1.07
	P01887	*B2m*	Beta-2-microglobulin#	119	1.07
	P08226	*Apoe*	Apolipoprotein E^*^	311	1.05
	P34884	*Mif*	Macrophage migration inhibitory factor#	115	1.01

*The UniprotKB code, gene symbol, description, length (amino acids), and cAMP-induced fold-change for each detected protein are shown*.

* Indicates concordance with Hansen (2014),

# *Indicates concordance with Ojima et al. (2016)*.

To ascertain whether secreted proteins underwent transcriptional regulation in response to cAMP, we measured the transcript levels of selected components of the distinct groups of differentially secreted proteins. We found that secreted proteins that were up-regulated by cAMP did not show a systematic relationship with transcript expression (Figure 1D). Among the ECM-related genes, the three tested collagen gene transcripts (*Col1a1, Col4a1*, and *Col4a2)* failed to show cAMP-induced up-regulation; indeed *Col1a1* was down-regulated. In contrast, the transcript level of *Lamb1* was increased by cAMP, consistently with the up-regulation of secreted laminin-β1 protein in cAMP-treated cultures. With respect to extracellular enzymes, the transcript levels of *Xdh* and *Ppia* were increased in response to cAMP, as observed for the corresponding secreted proteins. The analyzed adipokine-encoding gene transcripts varied: *Fabp4* was up-regulated, *Adipoq* was down-regulated, and *Rbp4* was unchanged. Varied changes were also observed for the transcripts encoding some of the other cAMP-induced secreted proteins: cAMP increased the transcript level of *Grn* (granulins), but decreased that of *Orm1* (orosomucoid). Among the transcripts encoding for secreted proteins whose abundance was reduced by cAMP treatment, we found a more consistent pattern: the transcript levels of *Efemp1, Lpl, Lbp*, and *B2m* were all significantly down-regulated in response to cAMP (Figure 1E).

Given the discordance between proteome-based detection of secreted proteins and the corresponding transcripts in some cases, levels of selected proteins from distinct functional groups were individually quantified. COL1A1, LAMB1, RBP4, and GRN proteins were confirmed to be up-regulated and LBP to be down-regulated by cAMP. However, adiponectin (ADIPOQ) and ORM1 were not confirmed to be up-regulated by cAMP, and results were more concordant with transcript changes (Figure 1F).

## Discussion

To our knowledge, this report describes the first direct assessment of the protein secretome of cultured brown adipocytes stimulated with cAMP, which triggers thermogenic activation. Thirteen of the proteins we detected with our iTRAQ-based method coincided with those identified by Hansen et al. (2014) using indirect signal sequence trap method to identify brown adipocyte-secreted proteins in response to norepinephrine (Table 1). They corresponded mainly to ECM-related factors (6) and the miscellaneous group of proteins (5). Moreover, a recent iTRAQ-based detection of proteins secreted early after induction of 3T3-L1 white adipocyte differentiation (Ojima et al., 2016) using isobutyl-methylxanthine (a phosphodiesterase inhibitor that raises intracellular cAMP) yielded results that were similar to our data. Matches were seen for 41% of the ECM-related proteins (12 of 29), 66% of the adipokines (4 of 6), and 60% of the “others” (6 of 10) (Table 1). This similarity between the two data sets may reflect the activation of common cAMP-dependent pathways in adipogenic cells.

A remarkable finding of our study is the strong representation (around 50%) of ECM-related components in the cAMP-induced secretome of brown adipocytes. A functional and flexible ECM is essential for healthy expansion of WAT (Sun et al., 2013). The thermogenic activation of BAT causes a tissue recruitment that involves hypertrophic and hyperplastic processes (Cannon and Nedergaard, 2004). The cAMP-induced secretion of ECM components, such as specific types of collagen and matricellular proteins, may be relevant to the remodeling of ECM under conditions of BAT expansion in response to thermogenic activation. Further research will be needed to explore this novel notion in BAT biology.

We identified several components of the complement system in the cAMP-induced secretome of brown adipocytes. Hansen et al. (2014) also identified several components of the complement system in their brown adipocyte secretome analysis. Interestingly, a transcriptomic analysis identified complement factor-4 as one of the top secreted protein-encoding transcripts up-regulated in human BAT vs. WAT (Svensson et al., 2011). Complement factor-D (adipsin), one of the first white adipokines identified (Cook et al., 1987), was here found to be up-regulated in the secretome of cAMP-treated brown adipocytes. Further research is warranted to ascertain the role of complement system components in the signaling that originates in brown adipocytes upon thermogenic activation.

Our study identified several brown adipokines previously reported to be regulated in response to thermogenic activation in adipose tissues, such as adiponectin (Hui et al., 2015), RBP4 (Rosell et al., 2012), angiotensinogen (Cassis, 1993), fatty acid binding protein-4 (Shu et al., 2017), and lipopolysaccharide binding protein (LBP) (Gavaldà-Navarro et al., 2016). These findings further validate our proteomic analysis. However, our secretome data did not include other known brown adipokines, such as IL-6, FGF21, or neuregulin-4 (NRG4). This is likely to reflect that such proteins are present at relatively low levels in the cell culture medium (1–10 ng/ml) (Burýsek and Houstek, 1997; Hondares et al., 2011; Rosell et al., 2014). In contrast, the levels of secreted proteins such as adiponectin or LBP exceed 100 ng/ml in the culture media of adipocyte culture systems (Díaz-Delfín et al., 2012; Moreno-Navarrete et al., 2013). The relatively low-level release of some secreted proteins by brown adipocytes may be a limitation in the ability to identify secretome components using proteomic analysis of culture media.

We found only partial concordance between the cAMP-regulated secreted proteins according to proteomics and the corresponding transcript levels. The discordance was particularly relevant for cAMP-induced ECM components and adipokines, whereas cAMP-repressed secreted factors showed more concordance. Moreover, individual quantification of a set of secreted protein candidates responsive to cAMP confirmed most, but not all, changes detected in the proteomics assays, even in cases when transcript levels were discordant. This suggests that post-transcriptional mechanisms are involved in the cAMP-mediated regulation of protein secretion by brown adipocytes and that transcriptomic approaches may therefore be limited in their ability to predict comprehensively the BAT secretome. Moreover, our data highlight the importance of specific validation of proteomics-based findings using individual quantification of secreted protein candidates.

In conclusion, the current study opens new venues for research of the secretome of brown adipocytes. Beyond helping direct further research on novel brown adipokine candidates, our work suggests two new research foci, namely: (a) understanding the importance of the synthesis of ECM components by brown adipocytes for BAT remodeling during thermogenic adaptation, and (b) determining the roles of the complement-related proteins by brown adipocytes upon thermogenic activation.

## Author Contributions

JV performed the brown adipocyte cell cultures, proteomic analytics, qRT-PCR, immunoblot, and ELISA assays. RC performed the bioinformatic analysis of secretability. MG and FV designed the study and compiled the data. FV and JV wrote the manuscript.

### Conflict of Interest Statement

The authors declare that the research was conducted in the absence of any commercial or financial relationships that could be construed as a potential conflict of interest.
