# Supplementary material for: Secretory Proteome of Brown Adipocytes in Response to cAMP-Mediated Thermogenic Activation
Source: Front Physiol. 2019 Feb 7;10:67. doi: 10.3389/fphys.2019.00067 (PMC6374321; doi:10.3389/fphys.2019.00067)
Supplement: Supplementary file 1 [file Table_1.DOCX]

Supplementary Material

Secretory proteome of brown adipocytes in response to thermogenic activation

Joan Villarroya et al.

*** Correspondence:**  Francesc Villarroya, fvillarroya@ub.edu

1. **Expanded methodology for iTRAQ analysis of brown adipocyte culture media**

**Protein digestion**

The protein contents of the sampled media were quantified using a Micro BCA™ Protein Assay Kit (Thermo Fisher Scientific, Waltham, MA, USA). For each sample, 200 μg of protein was digested with trypsin using the FASP (Filter-Aided Sample Prep) technique. The samples were diluted to 450 μL with 500 mM triethyl ammonium bicarbonate (TEAB)/8M urea, pH 8.0-8.5, further reduced with 5mM Tris (2-carboxyethyl) phosphine (60 min, 35ºC), and alkylated with 30mM iodoacetamide (21ºC, 20 min). An Amicon Ultra-15, membrane PLGC Ultracel-PL (10 kDa; Merck-Millipore, Burlington, MA, USA) and three rounds of centrifugation (12000 g, 30 min), wew used to remove interfering compounds. The samples were then washed with 300 μL of 500 mM TEAB, pH 8.0-8.5, and digested with sequence-grade modified trypsin (Promega; Madison, WI, USA) in the same buffer plus 1M urea (3.3 μg trypsin/sample; 37ºC, pH 8.5, 16h). The resulting peptide mixture was recovered by three rounds of centrifugation, washed with 200 μL 500 mM TEAB, dried (SpeedVac vacuum system, ThermoFisher Scientific) and stored at -20ºC.

**Peptide labeling**

Samples were resuspended in 200 μL 1% formic acid solution (FA), desalted, concentrated with a C18 microcolumn (top tip, PolyLC, Columbia, MD, USA) per the manufacturer's indications, and eluted with 200 μL of 70% acetonitrile (ACN)/0.1% FA. An aliquot of 100 ng was subjected to liquid chromatography-tandem mass spectrometry (LC-MSMS, see below) for determination of the per-sample peptide quantity prior to iTRAQ labeling. The rest of the sample was dried (SpeedVac), resuspended in 30 μL 500 mM TEAB, and subjected to iTRAQ labeling (iTRAQ™ 8plex Multiplex kit; Merck Sigma-Aldrich) according to the product specifications. Briefly, for each sample, 70 μl of ethanol was added to a vial of iTRAQ labeling reagents, and the mixture was vortexed for 1 min and then centrifuged. The contents of labeling vials were transferred to a sample tube, and the sample-iTRAQ mixture was incubated at room temperature for 2 h to allow the iTRAQ labeling reaction. The reaction mixture was diluted with 100 μl of water to quench the reaction, and an aliquot was analyzed by LC-MSMS to ensure complete labeling before sample mixing. Finally, the labeled samples were combined and labeled with the isobaric tag reporters as follows: C1/113; C2/114, C3/115, cAMP1/116, cAMP2/117, cAMP3/118. The combined iTRAQ-labelled sample was cleaned using C18 clean-up (reverse phase, toptip C18) (PolyLC, Columbia, MD, USA) and SCX cleanup (strong cationic exchange, P200 toptip, PolySULFOETHYL A) (PolyLC). In C18 clean-up step, samples were resuspended in 100 μL 1% FA solution, loaded to a C18 toptip, washed twice (50 μL 0.1% FA), eluted from the tip with 200 μL of 70% ACN/0.1% FA and dried in SpeedVac. Then, samples were resuspended in 100 μL in 20% ACN/0.1% FA (pH 2.7-3), loaded to a SCX P200 toptip, washed (3X 50 μL 20% ACN/0.1% FA) and finally eluted from the tip with 200 μL (4X 50 μL) 5% ammonium hydroxide/30%methanol. Finally, 100 μL of water (HPLC grade) was added to the samples and dried down in SpeedVac.

**LC-MSMS analysis**

Each dried peptide mixture was analyzed using a nanoAcquity liquid chromatograph (Waters; Milford, MA, USA) coupled to a LTQ-Orbitrap Velos mass spectrometer (Thermo Fisher Scientific). Peptides were trapped on a Symmetry C18TM trap column (5μm 180μm x 20mm), and separated using a C18 reverse phase capillary column (75 μm Øi, 25 cm, 1.7μm BEH column, nano Acquity) (Waters). The gradient used for peptide elution was as follows: from 2% to 35 % B over 155 minutes, and then from 35% to 45% over 20 min (A: 0.1% FA; B: 100% ACN/ 0.1%FA), at a flow rate of 250 nl/min.

The eluted peptides were subjected to electrospray ionization (PicoTipTM, New Objective, Woburn, MA, USA). Peptide masses (m/z 300-1800) were analyzed in the data dependent mode, where a full Scan MS in the Orbitrap with a resolution of 30,000 FWHM at 400m/z. The most abundant peptides (up to 10; minimum intensity, 2000 counts) were selected from each MS scan. They were then fragmented by HCD (higher energy collision dissociation) in the C-trap using nitrogen as the collision gas, with a 50% normalized collision energy, and analyzed in the Orbitrap with a resolution of 7,500 FWHM at 400m/z. The scan time settings were: full MS, 250 ms (1 microscan) and MSn, 300 ms (2 microscans). Generated .raw data files were collected with usin Thermo Xcalibur (v.2.1.0.1140) (Thermo Fisher Scientific).

**Database search**

Thermo Proteome Discover (v.1.3.0.339) (Thermo Fisher Scientific) was used to generate a single Mascot generic file (.mgf) from the .raw data files obtained in the MS analyses. This .mgf file was applied to perform a database search using the Mascot search engine against the SwissProt/Uniprot database. Both target and decoy database were searched to obtain a false discovery rate (FDR; <5%). From the database search, Mascot generated a .DAT file; this was used to elaborate the relative ratio of quantitation based on the reporter ions intensities.

**Quantitative analysis**

The IsobariQ analysis software (http://www.ncbi.nlm.nih.gov/pubmed/21067241) was used to perform relative quantitation of the proteins from our iTRAQ data. Only proteins with at least two different unique peptides were quantified. Peptide ratios were log transformed and subjected to median ratiometric normalization to avoid instrumental and methodological variability, which can decrease the precision and accuracy, respectively, of data. The protein ratio was calculated using the median of the quantified peptide ratios (or the mean for two quantified peptides). The observed ratios were C1(113)/cAMP1(116), C2(114)/cAMP2(117) and C3(115)/cAMP3 (118), which represented the first, second and third biological replicates, respectively. For each biological replicate, four LC-MSMS technical replicates were performed; thus we calculated the ratio of each protein using four replicate means. Outlier Grubb´s test was applied as appropriate during the data processing steps.

| **Gene name** | **Catalogue number** |
| --- | --- |
| *Adipoq* | Mm00456425_m1 |
| *B2m* | Mm00437762_m1 |
| *Col1a1* | Mm00801666_g1 |
| *Cox7a1* | Mm00438297_g1 |
| *Dio2* | Mm00515664_m1 |
| *Fabp4* | Mm00445878_m1 |
| *Grn* | Mm00433848_m1 |
| *Lbp* | Mm00493139_m1 |
| *Lpl* | Mm00434764_m1 |
| *Ppargc1a* | Mm01208835_m1 |
| *Ppia* | Mm02342430_g1 |
| *Rbp4* | Mm00803264_g1 |
| *Ucp1* | Mm01244861_m1 |
| *18S* | Hs99999901_s1 |

**2- TaqMan assays and primers used for SyberGreen analysis in qRT-PCR analysis of RNA transcript**

1. **TaqMan probes**
2. **Oligonucleotide primers used for SyberGreen analysis**

| **Gene name** | **Primers sequence** |
| --- | --- |
| *Col4a1* | Fw: TCCTCACTGTGGATCGGCT  Rv: TTGGATCAGGAGCGCCATTT |
| *Col4a2* | Fw: CGAGAGGCGTCTCTGGATTC  Rv: CGCCTTTTGAGATTACGCCG |
| *Efemp1* | Fw: CGCCAGTTCAGACCTACCAG  Rv: GTCAGCTGGGTTTCTTCGGA |
| *Lamb1* | Fw: CCGGGTGAGGAGAACAAAGT  Rv: TCCCAAAGTCAGAAGACCGC |
| *Xdh* | Fw: TCACGATGACGAGGACAACG  Rv: TTCAGCCTCAGCAACTCTGG |

**3- Antibody-based assays for specific detections of secreted proteins in culture medium of cAMP-treated versus non-treated cells**

| **Protein name** | **Company name** | **Reference number** |
| --- | --- | --- |
| Mouse collagen, type I, alpha 1 (COL1A1) ELISA kit | CUSABIO Technology (Houston, TX, USA) | CSB-EL005727MO-24T |
| Mouse Laminin subunit beta-1 (LAMB1) ELISA kit | CUSABIO Technology (Houston, TX, USA) | CSB-EL012730MO-24T |
| Adiponectin (ADIPOQ) Mouse ELISA Kit | Invitrogen (Thermo Fisher) Waltham, MA, US | KMP0041 |
| Retinol binding protein-4 antibody | ENZO Biochem. (Farmingdale, NY, USA) | ALX-210-974-0100 |
| Mouse Granulins (GRN) ELISA kit | CUSABIO Technology (Houston, TX, USA) | CSB-EL009939MO-24T |
| Mouse alpha1-Acid glycoprotein, alpha1-AGP (ORM1) ELISA kit | CUSABIO Technology (Houston, TX, USA) | CSB-E08581m-24T |
| Mouse Lipopolysaccharide-binding protein (LBP) ELISA kit | CUSABIO Technology (Houston, TX, USA) | CSB-EL012775MO-24T |
